# Electrochemical Behavior of Cobaltocene in Ionic Liquids

**DOI:** 10.1007/s10953-013-9957-1

**Published:** 2013-02-02

**Authors:** Andrzej Lewandowski, Lukasz Waligora, Maciej Galinski

**Affiliations:** Faculty of Chemical Technology, Poznań University of Technology, 60 965 Poznań, Poland

**Keywords:** Ionic liquid, Reference redox couple, Cobaltocenium

## Abstract

The electrochemical behavior of cobaltocenium has been studied in a number of room temperature aprotic ionic liquids. Well defined, diffusion controlled, anodic and cathodic peaks were found for the Cc^+^/Cc (cobaltocenium/cobaltocene) reduction/oxidation on gold, platinum and glassy carbon electrodes. Values of the peak separation parameters suggest quasireversibility or even irreversibility for the redox process. The difference between the ferrocene/ferrocenium and cobaltocenium/cobaltocene couples has been evaluated as equal to (1.350 ± 0.020) V. Values of the cobaltocenium (Cc^+^) diffusion coefficients *D* have been calculated on the basis of the Randles–Sevcik equation.

## Introduction

Room temperature ionic liquids [RTILs] may be used as solvents in many applications, as described in references [1–6], including electrochemistry [7–10]. In the latter case a reference system is necessary to compare potentials measured in different solvent-free RTILs. Different reference systems have been proposed, including pseudo-reference electrodes (metal immersed in an electrolyte, usually Pt or Ag), according to references [11, 12]. In such a case, the surface of the pseudo-reference system must be much larger in comparison to the working electrode; consequently, the current density at the reference is much lower. Half-cells based on the Ag|Ag^+^ or Ag|AgCl|Cl^−^ systems (where a salt, being a source of Ag^+^ or Cl^−^ ions, is dissolved in a mixture of acetonitrile with ionic liquid) have also been recommended as references for RTILs [13–15] and also for high temperature ILs (LiCl–KCl–CaCl_2_) [16]. Silver wire, immersed in a solution of silver(I) salt and cryptand 222 in molecular liquids [17] and ionic liquids [18], has been proposed as a stable reference system.

Finally, electrode potentials may be expressed versus an inner reference organometallic redox system, which consists of a large cation and its reduced form. The oxidized and reduced forms, both of large radius, may be assumed to be solvated similarly in different solvents. This leads directly to the assumption that redox potentials of such couples should be comparable in different solvents. Consequently, a given redox couple may be regarded as a universal potential reference. Organometallic redox couples such as bis(biphenyl)chromium(0)/(I) (BCr|BCr^+^), ferrocene|ferrocenium (Fc|Fc^+^), and cobaltocene|cobaltocenium (Cc|Cc^+^) have been investigated in various RTILs as described in the literature [10, 19–34], including a study on the applicability of cobaltocenium reduction as a reference for ionic liquids [30] and a detailed study on the simultaneous presence of both (Cc|Cc^+^) and (Fc|Fc^+^) couples in RTILs [32]. The general aim of the present study was to conduct a systematic investigation of the Cc/Cc^+^ redox reference system in various aprotic ionic liquids at different electrodes.

## Experimental

### Chemicals

Bis(cyclopentadienyl) cobalt(III) hexafluorophosphate (cobaltocenium hexafluorophosphate, ${\text{Cc}}^{ + } {\text{PF}}_{6}^{ - }$, Aldrich), silver perchlorate (AgClO_4_, Fluka), and cryptand 222 (4.7.13.16.21.24-hexaoxa-1.10-diazabicyclo [8.8.8] hexacosane, Merck) were used as purchased. Acetonitrile (AN, Merck) was distilled before use. Room temperature ionic liquids: *N*-methyl-*N*-propylpyrrolidinium bis(trifluoromethanesulfonyl)imide (MePrPyrrNTf_2_) (Iolitec), *N*-butyl-*N*-methylimidazolium triflate (BuMeImOTf) (Iolitec), *N*-butyl-*N*-methylpyrrolidinium bis(trifluoromethanesulfonyl)imide (BuMePyrrNTf_2_) (Iolitec), *N*-butyl-*N*-methylpyrrolidinium triflate (BuMePyrrOTf) (Merck), diethylmethylsulphonium bis(trifluoromethanesulfonyl)imide ([Et_2_MeS][NTf_2_] (Iolitec), and triethylsulphonium bis(trifluoromethanesulfonyl)imide ([Et_3_S][NTf_2_] (Iolitec) were used as purchased. *N*-ethyl-*N*-methylimidazolium dicyanoimide (EtMeImN(CN)_2_) was obtained according to a published procedure [35] by metathesis of EtMeImBr with AgN(CN)_2_ in an aqueous solution [36]. *N*-methyl-*N*-propylpiperidinium bis(trifluoromethanesulfonyl)imide (MePrPipNTf_2_) was obtained according to a method described in the literature [37].

#### Water Content and Purity

The water content in aprotic ionic liquids, analyzed with a standard Karl–Fisher titrant (HYDRANAL^®^ Composite 1, 1 mL/10 mg H_2_O), was below the detection limit. All of the RTILs were colorless. The purities of ionic liquids for electrochemical purpose were analyzed with cyclic voltammetry on platinum, gold and glassy carbon working electrodes. No reduction or oxidation peaks were detected between the anodic and cathodic decomposition potentials.

### Apparatus and Procedures

Voltammetric measurements were performed in a three-electrode arrangement. Working electrodes: Au (1.50 mm diameter, 1.77 mm^2^), Pt (1.50 mm, 1.77 mm^2^) and glassy carbon (3.00 mm diameter, 7.07 mm^2^) were disc shaped and sealed in poly(tetrafluoroethylene). Before measurements, the electrodes were polished with aluminum oxide paste in water (Al_2_O_3_, 150 mesh, Merck) and then washed with acetone. The counter electrode was a platinum sheet (0.5 × 1.0 cm). The reference electrode consisted of a silver wire immersed in a solution of AgClO_4_ (0.01 mol·dm^−3^) and cryptand 222 (0.1 mol·dm^−3^) in acetonitrile [17]. The reference electrode compartment was separated by a glass frit from the cell containing the ionic liquid. Preparation of the solutions, weighing of the samples, and cell assembly were performed in a glove-box under a dry argon atmosphere. Tested electrolytes were deaerated with argon for 30 min prior to measurements. Voltammetric curves were obtained with the μAutoLab Electrochemical System (Eco Chemie, The Netherlands) at (25 ± 0.1)  °C. The initial scan was carried out to more negative potentials (reduction of cobaltocene: Cc^+^ + e^−^ → Cc^0^) followed by the reverse anodic scan (oxidation of Cc^0^). Two reduction/oxidation scans were recorded in each case. The baseline of each neat ionic liquids was measured before experiments with Cc/Cc^+^ solutions. The ohmic resistance *R* between electrodes was determined from impedance spectra (using an *ac* impedance analyzer Atlas-Sollich, Poland), in the frequency range of 100 kHz to 1 Hz with 10 mV amplitude.

## Results and Discussion

### Electrolyte Conductivity and IR Ohmic Drop

The conductivity of the electrolyte influences the resistance between electrodes of the cell. In the case of ionic liquid electrolytes, the specific conductivity is typically between ca. 10 and 0.01 mS·cm^−1^, according to Ref. [8], which may lead to resistances differing by three orders of magnitude. The resistance was obtained by deconvolution of impedance spectra according to an equivalent circuit, consisting of resistance *R* in series with the Warburg impedance and charge transfer resistance, in parallel to the double layer capacity. The resistance *R*, determined from impedance spectra, was between ca. 200 Ω (Et_3_SNTf_2_) and 2,400 Ω (MePrPipNTf_2_). At a typical current level of 10 μA, the *IR* distortion of the potential was in the range of 0.2–24 mV.

### CV Curves

**Fig. 1:**
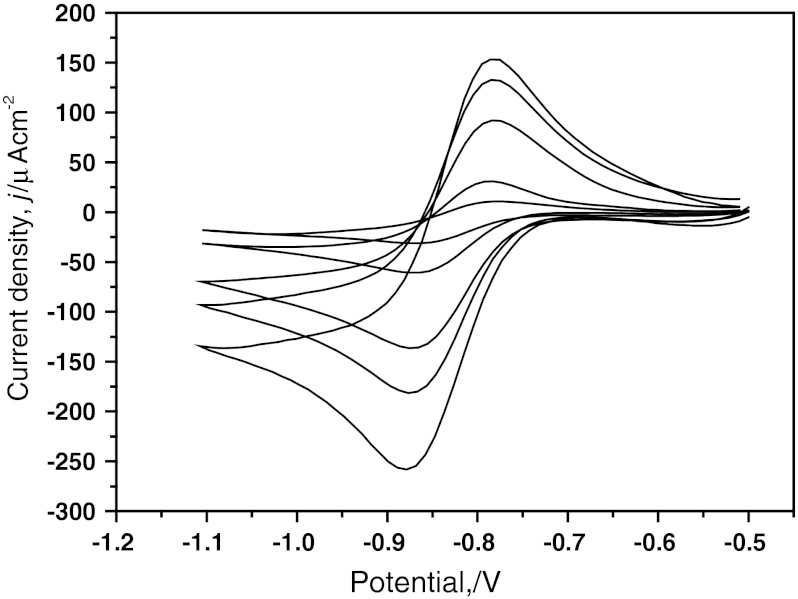
Cyclic voltammetry for cobaltocene Cc^+^/Cc in BuMeImOTf on a Pt electrode at different sweep rates (from 2 to 200 mV s^−1^); reference electrode: Ag/Ag^+^222 in acetonitrile

Figure 1 presents typical cv curves, after background current and *IR* drop corrections, for cobaltocenium/cobaltocene (Cc^+^/Cc) reduction/oxidation in BuMeImOTf ([${\text{CcPF}}_{6}^{ - }$] = 9.18 mmol·L^−1^) at potential sweep rates from (2 to 200) mV·s^−1^, versus the Ag|Ag^+^222, AN reference electrode. Potentials of the peak maximum, *E*_pa_ and *E*_pc_, for Cc^+^ cathodic reduction and Cc anodic oxidation were −854 and −762 mV, respectively. Similar CV curves were obtained for a number of ionic liquids as solvents recorded at the three different electrodes (Pt, Au, GC). Measurements of Δ*E*_1/2_ (Cc^+^/Cc^0^) over a period of 24 h gave stable values within ca. 1–2 mV, indicating no significant changes in the liquid junction potential between RTILs and the reference electrode electrolyte. Differences between the cathodic and anodic peak potentials, *E*_pa_−*E*_pc_, ½(*E*_pa_ + *E*_pc_), and *E*_p_−*E*_p/2_ values (*E*_p/2_ is the half-peak potential), and peak current densities *j*_pa_ and *j*_pc_, in the studied ionic liquids, are collected in Table 1. The difference between the cathodic and anodic peak potentials, *E*_pa_−*E*_pc_, is ≥81 mV, while the value predicted by the theory for reversible processes, according to Ref. [38], is (57–60) mV depending on the switching potential. The results indicate a quasi-reversible redox process for the cobaltocenium/cobaltocene couple. A similar behavior was found for the ferrocene|ferrocenium couple in a number of ionic liquids in our previous paper [34]. On the other hand, the *E*_p_−*E*_p/2_ values are close to 56 mV, typical of reversible processes [38]. In some protic RTILs, the *E*_p_−*E*_p/2_ values are close to the theoretical value of 56 mV, but in some cases they are higher (even as much as 70 mV). The formal potential ½(*E*_pa_ + *E*_pc_) for the Cc^+^/Cc couple in aprotic ionic liquids may be approximated by the value−(831 ± 13) mV (versus the Ag|Ag^+^222 in AN reference). Formal potentials obtained in this study for cobaltocene may be referred to potentials for ferrocene measured in a number of protic and aprotic ionic liquids [34]. Table 2 presents differences between formal potentials of ferrocene/ferrocenium and cobaltocenium/cobaltocene couples in ionic liquids as well as molecular liquids; these results were calculated from the ½(*E*_pa_ + *E*_pc_) values measured versus the cryptate electrode Ref. [34], or versus reference systems mentioned in references [15, 28–41]. Inspection of Table 2 shows that the *E*_1/2_(Fc/Fc^+^)−*E*_1/2_ (Cc^+^/Cc) difference may be approximated by the value (1.350 ± 0.020) V and is in agreement with values obtained by other authors [15, 32, 40, 41]. Such good agreement of the potential difference (±0.02 V) suggests that the solvation of both reference couples is nearly independent of the electrolyte.

**Table 1:** Electrochemical parameters for cobaltocene cyclic voltammetry in aprotic ionic liquids at platinum, gold and glassy carbon electrodes, with reference electrode Ag|Ag^+^ (0.01 mol·L^−1^) cryptand 222 + (0.1 mol·L^−1^) AN

Electrolyte/	Scan rate	*E*_pa_	*E*_pc_	Δ*E*_*p*_	½ (*E*_pa_ + *E*_pc_)	*E*_*p*_ – *E*_*p/2*_	*j*_pa_	*j*_pc_
Electrode	mV·s^−1^	V	V	V	V	V	μA·cm^−2^	μA·cm^−2^
MePrPyrrNTf_2_	2	−0.752	−0.864	0.112	−0.808	0.067	44	40
Pt	10	−0.753	−0.865	0.112	−0.809	0.052	63	71
(10.42 mmol·L^−1^)	50	−0.754	−0.867	0.113	−0.811	0.056	134	147
	100	−0.755	−0.878	0.123	−0.817	0.060	195	206
	200	−0.746	−0.879	0.133	−0.813	0.065	260	270
MePrPyrrNTf_2_	2	−0.763	−0.854	0.091	−0.809	0.057	45	94
GC	10	−0.764	−0.855	0.091	−0.810	0.061	100	96
(10.42 mmol·L^−1^)	50	−0.757	−0.859	0.102	−0.808	0.059	222	219
	100	−0.759	−0.861	0.102	−0.810	0.057	317	317
	200	−0.762	−0.875	0.113	−0.819	0.063	444	447
MePrPyrrNTf_2_	2	−0.762	−0.853	0.091	−0.808	0.059	39	43
Au	10	−0.773	−0.854	0.081	−0.814	0.058	91	93
(10.42 mmol·L^−1^)	50	−0.775	−0.865	0.090	−0.820	0.058	204	217
	100	−0.776	−0.866	0.090	−0.821	0.059	295	323
	200	−0.779	−0.866	0.087	−0.823	0.059	436	496
BuMeImOTf	10	−0.770	−0.888	0.118	−0.829	0.055	61	86
Au	50	−0.805	−0.887	0.082	−0.846	0.057	142	196
(9.18 mmol·L^−1^)	100	−0.800	−0.883	0.083	−0.842	0.053	200	252
	200	−0.803	−0.884	0.081	−0.844	0.051	261	367
BuMeImOTf	2	−0.772	−0.888	0.116	−0.830	0.061	36	36
GC	10	−0.774	−0.876	0.102	−0.825	0.062	83	72
(9.18 mmol·L^−1^)	50	−0.777	−0.879	0.102	−0.828	0.065	183	158
	100	−0.779	−0.883	0.104	−0.831	0.065	239	230
	200	−0.781	−0.897	0.116	−0.839	0.064	332	323
BuMeImOTf	2	−0.782	−0.873	0.091	−0.828	0.053	19	30
Pt	10	−0.783	−0.874	0.091	−0.829	0.063	66	63
(9.18 mmol·L^−1^)	50	−0.784	−0.876	0.092	−0.830	0.059	135	131
	100	−0.785	−0.877	0.092	−0.831	0.057	182	184
	200	−0.785	−0.878	0.093	−0.832	0.054	242	267
BuMePyrrNTf_2_	2	−0.751	−0.865	0.114	−0.808	0.059	22	22
Au	10	−0.761	−0.865	0.104	−0.813	0.060	53	51
(6.74 mmol·L^−1^)	50	−0.772	−0.867	0.095	−0.820	0.060	122	117
	100	−0.772	−0.868	0.096	−0.820	0.064	182	184
	200	−0.773	−0.870	0.097	−0.822	0.067	260	265
BuMePyrrNTf_2_	2	−0.752	−0.843	0.091	−0.798	0.060	21	22
GC	10	−0.753	−0.844	0.091	−0.799	0.060	49	49
(6.74 mmol·L^−1^)	50	−0.754	−0.845	0.091	−0.800	0.060	104	105
	100	−0.755	−0.856	0.101	−0.806	0.057	148	143
	200	−0.756	−0.857	0.101	−0.807	0.066	192	198
BuMePyrrNTf_2_	2	−0.762	−0.843	0.081	−0.803	0.056	17	20
Pt	10	−0.763	−0.833	0.070	−0.798	0.058	44	45
(6.74 mmol·L^−1^)	50	−0.764	−0.844	0.080	−0.804	0.057	95	108
	100	−0.765	−0.845	0.080	−0.805	0.057	135	132
	200	−0.766	−0.845	0.079	−0.806	0.055	185	176
BuMePyrrOTf	10	−0.782	−0.885	0.103	−0.834	0.051	42	75
Au	50	−0.784	−0.887	0.103	−0.836	0.051	105	160
(10.8 mmol·L^−1^)	100	−0.786	−0.889	0.103	−0.838	0.051	161	236
	200	−0.789	−0.891	0.102	−0.840	0.056	240	320
BuMePyrrOTf	2	−0.784	−0.864	0.080	−0.824	0.057	28	27
GC	10	−0.785	−0.886	0.101	−0.836	0.058	64	59
(10.8 mmol·L^−1^)	50	−0.779	−0.880	0.101	−0.830	0.066	140	135
	100	−0.781	−0.895	0.114	−0.838	0.067	190	207
	200	−0.778	−0.902	0.124	−0.840	0.068	329	361
BuMePyrrOTf	2	−0.752	−0.864	0.112	−0.808	0.061	14	12
Pt	10	−0.763	−0.877	0.114	−0.820	0.050	45	50
(10.8 mmol·L^−1^)	50	−0.753	−0.901	0.148	−0.827	0.059	139	131
	100	−0.754	−0.915	0.161	−0.835	0.06	150	200
	200	−0.753	−0.921	0.168	−0.837	0.055	197	268
Et_2_MeSNTf_2_	2	−0.792	−0.873	0.081	−0.833	0.055	43	51
Au	10	−0.792	−0.877	0.085	−0.835	0.059	101	112
(10.21 mmol·L^−1^)	50	−0.783	−0.875	0.092	−0.829	0.059	237	254
	100	−0.784	−0.876	0.092	−0.830	0.059	336	372
	200	−0.785	−0.877	0.092	−0.831	0.057	473	534
Et_2_MeSNTf_2_	2	−0.783	−0.874	0.091	−0.829	0.059	50	51
GC	10	−0.784	−0.875	0.091	−0.830	0.056	113	118
(10.21 mmol·L^−1^)	50	−0.785	−0.877	0.092	−0.831	0.059	262	262
	100	−0.787	−0.879	0.092	−0.833	0.059	378	372
	200	−0.789	−0.882	0.093	−0.836	0.058	475	542
Et_2_MeSNTf_2_	2	−0.782	−0.873	0.091	−0.828	0.061	50	51
Pt	10	−0.783	−0.874	0.091	−0.829	0.057	93	95
(10.21 mmol·L^−1^)	50	−0.784	−0.875	0.091	−0.830	0.056	196	207
	100	−0.785	−0.876	0.091	−0.831	0.056	313	347
	200	−0.786	−0.877	0.091	−0.832	0.055	370	412
Et_3_SNTf_2_	10	−0.784	−0.87	0.086	−0.827	0.054	84	105
Au	50	−0.784	−0.87	0.086	−0.827	0.057	200	229
(6.12 mmol·L^−1^)	100	−0.785	−0.87	0.085	−0.828	0.057	309	347
	200	−0.785	−0.869	0.084	−0.827	0.057	433	475
Et_3_SNTf_2_	2	−0.772	−0.863	0.091	−0.818	0.060	46	42
GC	10	−0.773	−0.864	0.091	−0.819	0.057	98	102
6.12 mmol·L^−1^)	50	−0.774	−0.866	0.092	−0.820	0.057	216	218
	100	−0.776	−0.867	0.091	−0.822	0.059	319	319
	200	−0.777	−0.869	0.092	−0.823	0.060	441	459
Et_3_SNTf_2_	2	−0.773	−0.862	0.089	−0.818	0.063	41	41
Pt	10	−0.783	−0.863	0.080	−0.823	0.060	88	88
6.12 mmol·L^−1^)	50	−0.774	−0.864	0.090	−0.819	0.061	192	198
	100	−0.775	−0.864	0.089	−0.820	0.060	273	289
	200	−0.776	−0.864	0.088	−0.820	0.061	366	409
EtMeImN(CN)_2_	2	−0.782	−0.894	0.112	−0.838	0.060	111	111
Au	10	−0.792	−0.894	0.102	−0.843	0.065	383	445
(9.49 mmol·L^−1^)	50	−0.792	−0.895	0.103	−0.844	0.061	417	475
	100	−0.792	−0.895	0.103	−0.844	0.061	588	636
	200	−0.802	−0.883	0.081	−0.843	0.060	845	897
EtMeImN(CN)_2_	2	−0.782	−0.893	0.111	−0.838	0.069	127	136
GC	10	−0.782	−0.894	0.112	−0.838	0.056	227	280
(9.49 mmol·L^−1^)	50	−0.793	−0.885	0.092	−0.839	0.060	319	348
	100	−0.794	−0.885	0.091	−0.840	0.059	575	633
	200	−0.795	−0.887	0.092	−0.841	0.063	1002	987
EtMeImN(CN)_2_	2	−0.792	−0.883	0.091	−0.838	0.072	107	99
Pt	10	−0.792	−0.883	0.091	−0.838	0.068	190	207
(9.49 mmol·L^−1^)	50	−0.803	−0.883	0.080	−0.843	0.065	369	441
	100	−0.803	−0.884	0.081	−0.844	0.065	511	587
	200	−0.803	−0.884	0.081	−0.844	0.064	689	789
MePrPipNTf_2_	2	−0.762	−0.844	0.082	−0.803	0.058	21	30
Au	10	−0.763	−0.855	0.092	−0.809	0.061	66	68
(10.45 mmol·L^−1^)	50	−0.764	−0.858	0.094	−0.811	0.061	146	159
	100	−0.764	−0.871	0.107	−0.818	0.062	205	240
	200	−0.759	−0.892	0.133	−0.826	0.059	278	295
MePrPipNTf_2_	2	−0.773	−0.876	0.103	−0.825	0.057	30	44
GC	10	−0.755	−0.847	0.092	−0.801	0.060	61	62
(10.45 mmol·L^−1^)	50	−0.761	−0.853	0.092	−0.807	0.061	151	147
	100	−0.754	−0.868	0.114	−0.811	0.069	218	216
	200	−0.792	−0.9	0.108	−0.846	0.068	384	354

**Table 2:** A comparison of formal potentials of cobaltocene and ferrocene in ionic liquids (this study and a previous paper or literature results)

Electrolyte	Electrode^a^	*E*_1/2_(Fc/Fc^+^)−*E*_1/2_ (Cc^+^/Cc)/V
BuMeImBF_4_	Au, GC	1.345 [32]
BuMeImPF_6_	Au, GC	1.345 [32]
EtMeImBF_4_	Au	1.339 [31]
EtMeImBF_4_	Pt	1.333 [31]
EtMeImBF_4_	GC	1.336 [31]
MePrPyrrNTf_2_	Pt	1.325 this study and [34]
MePrPyrrNTf_2_	GC	1.327 this study and [34]
BuMeImOTf	Au	1.362 this study and [34]
BuMePyrrNTf_2_	Au	1.363 this study and [34]
BuMePyrrNTf_2_	Pt	1.333 [15]
BuMePyrrOTf	GC	1.347 this study and [34]
Et_2_MeSNTf_2_	Au	1.348 this study and [34]
Et_3_SNTf_2_	Au	1.354 this study and [34]
EtMeImN(CN)_2_	GC	1.332 this study and [34]
EtMeImN(CN)_2_	Pt	1.355 this study and [34]
MePrPipNTf_2_	Au	1.362 this study and [34]
EtMeImNTf_2_	Au. Pt. GC	1.330 [28]
MeImSBuNTf_2_	Au. Pt. GC	1.340 [28]
THF	Pt	1.400 [39]
DCM	Pt	1.390 [39], 1.351 [40]
AN	Pt	1.390 [39], 1.350 [41]

*AN* acetonitrile, *DCM* dichloromethane, *THF* tetrahydrofurane

### Diffusion

**Fig. 2:**
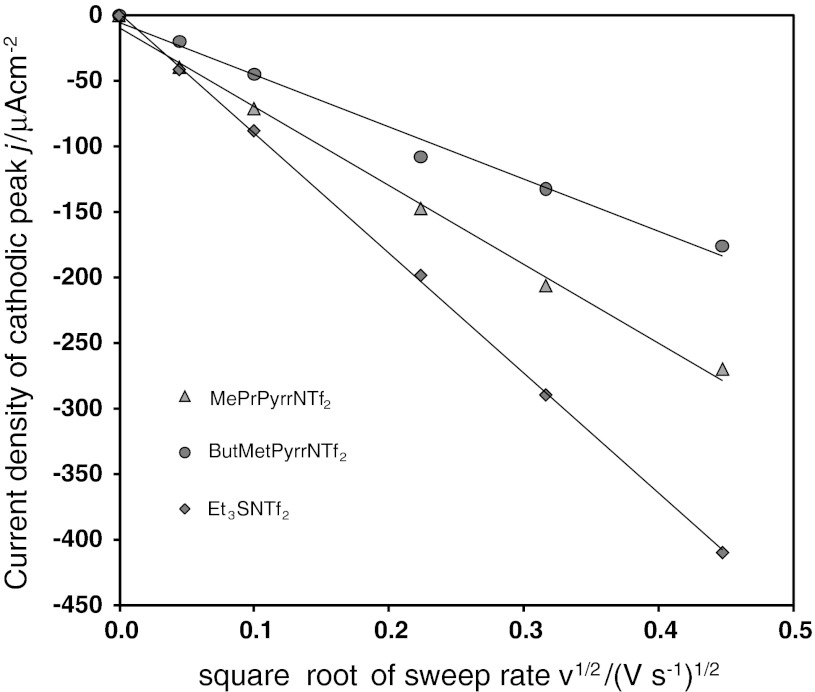
Dependence of the cathodic current peak density on the square root of sweep rate for cobaltocene reduction in ionic liquids: *black triangle*, MePrPyrrNTf_2_; *black circle*, BuMePyrrNTf_2_; *black diamond*, Et_3_SNTf_2_

Diffusion coefficients of the cobaltocenium cation were calculated from the Randles–Sevcik equation for a one-electron reduction:

1 $$ \left| {j_{\text{pc}} } \right| = 0.4463\;\left( {\frac{{F^{3} }}{R\;T}} \right)^{1/2} \;\left[ {{\text{Cc}}^{ + } } \right]\;\;D^{1/2} \;\nu^{1/2} $$

where *F* is the Faraday constant, *R* is the gas constant, *T* is the temperature, [Cc^+^] is the cobaltocenium bulk concentration, *D* stands for the Cc^+^ diffusion coefficient, and *v* is the sweep rate. Figure 2 presents examples of the cathodic peak current density as a function of square root of the sweep rate. Such a linear *j*_pc_ = *f*($\sqrt v$) function was observed in all of the ionic liquids. Values of the cobaltocenium diffusion coefficients, collected in Table 3, show that in the case of viscous aprotic RTILs the diffusion coefficients are of the order of (10^−6^ or 10^−7^) cm^2^ **·**s^−1^. Diffusion coefficients are between 0.52 × 10^−7^ cm^2^ **·**s^−1^ and 5.2 × 10^−7^ cm^2^ **·**s^−1^. Recently, three ionic liquids (EtMeImNTf_2_, BuMePyrrNTf_2_ and BuMeImPF_6_) were studied by cyclic voltammetry and rotating disc voltammetry [30]. The diffusion coefficient obtained here for BuMePyrrNTf_2_ is somewhat lower, ca. 0.72 × 10^−7^ cm^2^ **·**s^−1^, in comparison with 1.29 × 10^−7^ cm^2^ **·**s^−1^ reported in Ref. [30]. Values reported for other RTILs are *D*(EtMeImNTf_2_) = 3.27 × 10^−7^ cm^2^ **·**s^−1^ and *D*(BuMeImPF_6_) = 3.73 × 10^−8^ cm^2^·s^−1^ [30]. Recently the diffusion coefficient of Cc^+^ in BuMeImBF_4_ and BuMeImPF_6_ was reported [32] to be slightly concentration sensitive. Values of *D*(Fc) in aprotic RTILs are usually of the order of (10^−8^–10^−7^) cm^2^ **·**s^−1^, similar to *D*(Cc^+^). Diffusion coefficients of the ferrocene/ferrocenium and cobaltocenium/cobaltocene couples have also been reported for allyl substited pyrrolidinium, piperidinium and morpholinium-based ionic liquids [33]. Diffusion coefficient values are in the same range of (10^−7^–10^−6^) cm^2^ **·**s^−1^, depending on IL’s viscosity.

**Table 3:** Cobaltocenium (Cc^+^) diffusion coefficients *D* (cm^2^ s^−1^) in ionic liquids, the ionic liquid viscosity *η* together with the *Dη* product

Ionic liquid	Electrode	*D* (Cc^+^) cm^2^·s^−1^ × 10^7^	*η* cP	*Dη* cm·g·s^−2^ × 10^7^
MePrPyrrNTf_2_	Au, GC, Pt	1.09	59 [42]	0.64
BuMeImOTf	Au, GC, Pt	0.80	90 [4]	0.72
BuMePyrrNTf_2_	Au, GC, Pt	0.72	85 [43]	0.61
BuMePyrrOTf	Au, GC, Pt	0.52	158 [48]	0.82
Et_2_MeSNTf_2_	Au, GC, Pt	1.64	36 [5]	0.59
Et_3_SNTf_2_	Au, GC, Pt	3.74	33 [47]	1.23
EtMeImN(CN)_2_	Au, GC, Pt	5.21	21 [44]	1.09
MePrPipNTf_2_	Au, GC, Pt	0.58	141 [42]	0.82

According to the Stokes–Einstein equation, Eq. 2, the main factor influencing the diffusion coefficient is the medium’s viscosity, *η*,

2 $$ D\eta = \frac{{k_{\text{B}} T}}{{6{{\uppi}}r}}, $$

where *k*_B_ is the Boltzmann constant and *r* is the cobaltocenium radius.

Table 3 shows the *Dη* values calculated on the basis of literature data on ionic liquid viscosities published in references [5, 42–48], which correspond to the Walden product. Inspection of Table 3 suggests that the *Dη* product may be approximated by the value (0.80 ± 0.4) × 10^−7^ cm**·**g**·**s^−2^. This result may also suggest that the cobaltocenium radius is constant and independent of the medium. An interesting aspect is a comparison (ratio) of diffusion coefficients of both popular metallocecenes, ferrocene and cobaltocenium, used as electrode potential references, *D*(Fc)/*D*(Cc^+^). Here, the solvation of both forms may be different due to the fact that ferrocene is a neutral molecule, while cobaltocenium is a cation. A comparison of *D*(Cc^+^) values (Table 3) with the corresponding *D*(Fc) literature values shows that *D*(Fc)/*D*(Cc^+^) > 1. For example, according to Ref. [31], the *D*(Fc)/*D*(Cc^+^) ratio is 3.0 in EMImBF_4_ {*D*(Fc) = 2.70 × 10^−7^ cm^2^ **·**s^−1^}, while the corresponding value in EMImNTf_2_, reported in Ref. [30] is 1.39 {*D*(Fc) = 3.27 × 10^−7^ cm^2^ **·**s^−1^}. For comparison, the *D*(Fc)/*D*(Cc^+^) ratio is also higher than unity in solutions with molecular solvents. For example *D*(Fc)/*D*(Cc^+^) = 1.77 in acetonitrile {*D*(Fc) = 2.3 × 10^−5^ cm^2^·s^−1^ [49] and *D*(Cc^+^) = 1.30 × 10^−5^ cm^2^·s^−1^ [39]}. All of this information suggests that the Fc molecule and Cc^+^ cation, although of similar shape and radius, are probably solvated differently, as suggested in Ref. [50]. The cobaltocenium cation may interact with anions present in the electrolyte and therefore have a higher effective radius and, hence, a somewhat lower diffusion coefficient. In general, diffusion coefficients of large organic compounds determined in RTILs are on the order of 10^−7^ cm^2^ **·**s^−1^ which is two orders of magnitude lower than in conventional molecular solvents [51].

## Conclusions

1.: Well-defined, diffusion controlled, anodic and cathodic peaks were found for the Cc^+^/Cc redox couple in a number of RTILs
2.: Values of *E*_p_−*E*_p/2_ are close to 56 mV, typical of reversible processes. On the other hand, the difference between cathodic and anodic peak potentials, *E*_pa_−*E*_pc_, is ≥81 mV, while the value predicted by theory for reversible processes is 57–60 mV, which indicates a quasi-reversible redox process
3.: The *E*_1/2_(Fc/Fc^+^)−*E*_1/2_ (Cc^+^/Cc) difference may be approximated by the value (1.350 ± 0.020) V
4.: Values of the cobaltocenium (Cc^+^) diffusion coefficients *D* are in the range of 0.5 × 10^−7^ cm^2^ **·**s^−1^–5.2 × 10^−7^ cm^2^ **·**s^−1^, depending on the medium’s viscosity *η*. The *Dη* value, corresponding to the Walden product, falls within the narrow range of 0.61 × 10^−7^ cm**·**g**·**s^−2^–1.23 × 10^−7^ cm**·**g**·**s^−2^
